# Epimedin C enhances mitochondrial energy supply by regulating the interaction between MIC25 and UBC in rodent model

**DOI:** 10.1371/journal.pone.0325031

**Published:** 2025-05-28

**Authors:** Mi Huang, Lei Yu, Zhong Li, Ying Wang, Chunlin Yang

**Affiliations:** 1 Orthopedics department, Wuhan Hospital of Traditional Chinese and Western Medicine, Wuhan, Hubei, China; 2 College of Pharmacy, Hubei University of Chinese Medicine, Wuhan, Hubei, China; 3 Endocrinology department, Wuhan Red Cross Hospital, Wuhan, Hubei, China; 4 State Key Laboratory of Plant Diversity and Specialty Crops, Guangdong Provincial Key Laboratory of Applied Botany, Guangdong Provincial Key Laboratory of Digital Botanical Garden, South China Botanical Garden, Chinese Academy of Sciences, China; Tianjin Medical University Cancer Institute and Hospital: Tianjin Medical University Cancer Institute & Hospital, CHINA

## Abstract

The study investigates the molecular mechanisms underlying the skeletal muscle-enhancing effects of Epimedin C, a natural flavonoid, focusing on its interaction with the mitochondrial cristae structural protein MIC25. Using C57BL/6 mice, we demonstrate that Epimedin C enhances exercise performance through preservation of mitochondrial function. Proteomic analysis identified MIC25 as a key protein modulated by Epimedin C, whose stability is regulated via ubiquitin-dependent degradation. Functional experiments revealed that Epimedin C disrupts the interaction between MIC25 and ubiquitin-conjugating enzyme C (UBC), preventing MIC25 degradation and maintaining the integrity of the mitochondrial contact site and cristae organizing system (MICOS). This stabilization preserves mitochondrial cristae structure, improves ATP production, and delays muscle fatigue. Notably, MIC25 overexpression mimicked Epimedin C’s effects, while its knockdown abolished these benefits. Our findings establish MIC25 as a critical effector of Epimedin C, elucidating a novel pathway through which flavonoids maintain mitochondrial homeostasis to enhance muscle function. These insights hold promise for developing therapies targeting muscle atrophy and metabolic disorders.

## Introduction

The structural integrity of mitochondria directly governs the persistence and relative magnitude of their energy supply capacity [1,2]. This functional characteristic is fundamentally sustained by cristae architecture, where specialized membrane proteins establish the structural foundation for mitochondrial inner membrane folding [3]. These proteins facilitate enzyme anchoring and provide essential mechanical support for respiratory chain complexes and energy metabolism-related machinery [4]. As evolutionarily conserved organelles of bacterial origin, mitochondria perform indispensable roles in cellular physiology, most notably through ATP generation via oxidative phosphorylation systems [5,6]. Moreover, they serve as critical signaling hubs coordinating metabolic regulation, cellular differentiation programs, and apoptotic pathways [7]. The mitochondrial network maintains dynamic plasticity through continuous fission-fusion cycles, a process tightly regulated by cellular signaling cascades and energy demands [8]. Their unique membrane topology, comprising four distinct sub-compartments – the outer membrane (OM), intermembrane space (IMS), inner membrane (IM), and matrix – preserves molecular evidence of endosymbiotic evolution [9–11].

The mitochondrial contact site and cristae organizing system (MICOS), embedded within the inner mitochondrial membrane (IMM), plays an essential role in cristae morphogenesis and structural maintenance. At the molecular level, the MICOS complex is fundamentally organized by conserved members of the MIC protein family. Mic25 initially emerged as a stress-responsive biomarker through genomic screening, exhibiting transcriptional downregulation following genotoxic insult [12]. This 26.5 kDa transmembrane protein, predominantly localized to the IMM, shares architectural homology with Mic19 – both contain an N-terminal myristoylation motif, a central DUF737 domain, and a C-terminal CHCH domain featuring dual CX9C motifs. However, despite these structural parallels, Mic25 demonstrates distinct functional properties compared to its paralog [12]. Genetic ablation studies reveal that Mic25 depletion induces mitochondrial elongation without significantly perturbing cristae ultrastructure or altering stoichiometry of other MICOS/MIB complex subunits [13]. Recent advances, however, suggest partial cristae remodeling upon Mic25 knockdown, characterized by reduced cristae junction density and impaired membrane connectivity [14]. Current structural models propose that Mic60, Mic19, and Mic25 constitute the tripartite core of MICOS (migrating at ~450–500 kDa in native gels), serving as an organizational scaffold for peripheral complex components to assemble the fully functional MICOS machinery [15].

Epimedin C, a bioactive flavonoid glycoside originally isolated from *Epimedium* species, exhibits dual immunomodulatory and antineoplastic properties [16,17]. This phytochemical has been traditionally employed in herbal medicine for renal tonification, musculoskeletal reinforcement, and physical performance enhancement [18–20]. Contemporary pharmacological studies confirm its potent anti-osteoporotic effects through mechanisms involving osteoblast activation and osteoclast inhibition [19,21]. In hepatocellular carcinoma models, Epimedin C demonstrates antiproliferative efficacy via coordinated modulation of cell cycle regulators – suppressing oncogenic drivers (c-Myc, cyclin D1, c-FOS) while upregulating CDK inhibitors (p21, p27) [18]. Furthermore, it reverses hydrocortisone-induced immunosuppression in murine models by stimulating lymphocyte proliferation and enhancing interleukin-2 (IL-2) production [18]. Notably, the compound enhances exercise endurance capacity through skeletal muscle functional optimization, as evidenced by improved motor performance in supplemented animal cohorts [22]. Intriguingly, despite these pleiotropic effects, the precise molecular mechanisms underlying Epimedin C-mediated skeletal muscle remodeling remain elusive.

In this investigation, we focus on MIC25, a mitochondrial structural protein exhibiting differential expression following early-phase Epimedin C intervention. Through integrated animal experimentation and cytological profiling, we systematically elucidate potential regulatory networks linking phytochemical exposure to mitochondrial architecture modulation.

## Materials and methods

### Animals and ethics statement

Four-week-old healthy c57bl/6 mice (SYXK (E) 2017−0067), male, weighing 20 ± 2g, total of 24 mice were purchased from Liaoning Changsheng Biotechnology Corporation and raised in Hubei University of Traditional Chinese Medicine. The experimental process and treatment flow of experimental animals are subject to the requirements of the experimental animal committee of Hubei University of Traditional Chinese Medicine. 24 c57bl/6 male mice were randomly divided into two groups, with 12 mice in each model group and test group. SPSS software generated random numbers from 1 to 24, and the animals were housed in cages with four animals each.

All animal experiments were performed in strict accordance with the principles of the Declaration of Helsinki. Approval was obtained from the Animal Welfare and Research Ethics Committee under protocols that were approved by the Hubei Provincial Center for Medical Experimental Animals (No.202210210).

Mice were housed in a pathogen-free facility under controlled conditions (temperature: 22 ± 1°C; humidity: 50 ± 10%; 12-hour light/dark cycle) with ad libitum access to standard chow and water. All efforts were made to minimize animal suffering, including the use of anesthesia (e.g., isoflurane or ketamine/xylazine) for surgical procedures and analgesics for post-operative pain management.

For terminal experiments, euthanasia was performed via [CO₂ asphyxiation/cervical dislocation under anesthesia/decapitation] followed by confirmation of death, in compliance with the AVMA Guidelines for the Euthanasia of Animals (2020). The sample size was determined statistically to ensure meaningful results while minimizing the number of animals used, in alignment with the “3Rs” principle (Replacement, Reduction, Refinement).

### Chemicals and reagent

Epimedin C (MCEChem, HY-N0260), D-luciferin potassium salt (Gold Biotech, LUCK-1G), RNeasy Micro Kit (Qiagen, 74106), SuperScript™ IV Reverse Transcriptase (Invitrogen, 18090010), Real-time PCR Master Mix (Toyobo, QPK-101), NE-PER™ Nuclear and Cytoplasmic Extraction Reagents (Thermo Scientific, 78333), Pierce™ Direct Magnetic IP/Co-IP Kit (Thermo Scientific, 88828), ECL Luminescent Solution (Thermo Scientific, 32209), Ni-NTA Agarose Beads (Invitrogen, R90101, MAN0017121), MitoTracker™ Green FM (Invitrogen, M46750), MitoTracker™ (Thermo Scientific, M7514), ATP Assay Kit (Med Chem Express, MCE, HY-K0314-100 T), MitoCheck Complex I Activity Assay Kit (Cayman Chemical, 700930), Mitochondrial Respiratory Complex V Staining Kit (Biotech Arbitrary, MBS9719098), ATP5MF Antibody (OriGene Technologies, AP20619PU-N)

### Exercise training

Treadmill test: Mice ran daily at 10 m/min for 10 min (10 days), preceded by 4-hour food/water deprivation. Physically abnormal individuals were excluded.

Swimming pool: Mice swam in 0.1 m/sec water flow for 10 min/day (10 days). A 5 ml CO₂ cylinder nozzle injected tail bubbles every 2 min to induce startling/acceleration. Pre-swim fasting matched treadmill protocols.

For the determination of Epimedin C dangerous concentration, take testosterone as the index[23], set different feeding dose gradients, and finally determine the safe concentration as 10ug/kg. The results of the testosterone determination are shown in Supplementary Fig 1.

### Explosive power tests

The swimming pool was set to 10 m/min water flow (80 cm depth). Bubbles were blown at mice tails every 30 seconds to induce convulsions. Thrust swimming counts were recorded until exhaustion.

### Endurance test

Mice ran on a treadmill at 10 m/min. Time was recorded from start to exhaustion.

### Material sampling

Gastrocnemius muscles from model group mice were dissected (tendons removed). Muscle tissue was used for protein extraction/mass spectrometry. Fresh slow-twitch muscles underwent electrophysiological testing to measure energy output waveforms.

### Tissue preparation & stimulation

Muscles with tendons were placed in oxygen-saturated medium (37°C). Oxygen supply was reduced pre-stimulation; liquid surface vibration ≤ instrument noise defined baseline (0 points). Tendons were mounted on metal hooks, and muscles were suspended. Microcurrent pulses (4/sec) induced contractions. Force and uniform force periods per pulse were recorded until contractions ceased.

### Euthanasia

Mice were euthanized via cervical dislocation using USP medical (>99.2%), bone-dry (>99.9%), or industrial (>99.0%) CO₂, per guidelines [24].

### Cell isolation & culture

C57BL/6 skeletal muscle was homogenized in collagenase II (0.5 mg/ml, 37°C, 30 min), filtered (70μm), and centrifuged (500g, 5 min). Sediment was resuspended in DMEM +10% FBS (1% p/s), plated, and incubated (1–2 hours). Non-adherent cells were transferred to new plates. Medium was replaced with 2% FBS DMEM after 24 hours. Slender muscle fiber morphology was observed post-3 days.

### Cell lines

Human skeletal muscle cells (Prosai, CP-H095) were cultured in DMEM +10% FBS at 37°C, with medium changes every 48 hours. A Trans-well chamber ensured directional muscle fiber alignment. Post-inoculation, cells adhered for 8 hours before eutrophic medium replacement. Uniformly arranged cells underwent IF staining.

### IF staining

Cells were PBS-washed (×2), stained with 1/10,000 diluted mitochondrial dye (KBM-2 medium) for 30 min (37°C, dark), washed (×3, 2 min each), and imaged under fluorescence microscopy (Table 1).

**Table 1:** Primers used in this work.

Name	Primer	Sequence
MIC12	Forward	ATGGGGAGCACGGAGAGCAGCGAG
	Reverse	GCCCTTGTGGGCGGCGCTCACGCA
APOO	Forward	ATGTTCAAGGTAATTCAGAGGTCCGT
	Reverse	CTTAGTTCCAGGTGAATTCTTCACATTTC
CHCHD3	Forward	ATGGGTGGGACCACCAGCACCCGCCGGG
	Reverse	CTCCCTTCTCAAGCATGCTCTGTGGCAT
IMMT	Forward	ATGCTGCGGGCCTGTCAGTTATCGGGTGTG
	Reverse	CTCTGGCTGCACCTGAGTGGTTCCTATT
PPP1CA	Forward	ATGTCCGACAGCGAGAAGCTCAACCTGGAC
	Reverse	TTTCTTGGCTTTGGCGGAATTGCGGGGTGG
UBC3	Forward	ATGTCGGGGATCGCCCTCAGCCGCCTTGCG
	Reverse	TGAGGGGGCAAACTTCTTCGCTGTGCTCG

### Statistical analysis

Data (mean ± SD) were analyzed using SPSS 21.0. Normal-distributed data underwent one-way ANOVA; non-normal data used nonparametric tests. P < 0.05 indicated significance.

### Strains & vectors

*E. coli* DH5α/BL21(DE3) (Transgene: CD201–01/CD801–02) and yeast (AH109/Y187; Clontech 630444/630457) were used. Vectors: pET28a, pGEX6p-1, pcDNA3.1, PRT BD, pgwb435 (CAS), and AAV9 (Addgene).

### Protein extraction

Muscle tissue was ground (liquid nitrogen, quartz sand), lysed (3 × volume buffer: 50 mM NaCO3, 50–100 mM NaCl, 5 mM DTT, 0.5–2% vitamin C, 0.029% NaN3, pH 8.0), ice-shaken (1 h), centrifuged (8000 rpm, 5 min), and supernatant analyzed via mass spectrometry within 24 h.

### Pull-down screening

His-tagged MIC25 (bait) was mixed with Ni-NTA beads (1000 μl, ice, 30 min), split into two tubes, washed (1X buffer), and processed per Invitrogen protocol (MAN0017121).

### Isoelectric Focusing (IEF)

24 cm pH 4–7 IPG strips were used. IEF parameters:

- Step 1: 30V, 6h
- Step 2: 60V, 6h
- Step 3: 200V, 1h
- Step 4: 500V, 2h
- Step 5: 1000V, 2h
- Step 6: 3500V, 2.5h
- Step 7: 10000V, 1h
- Step 8: 10000V, 8h
- Step 9: 500V, 12h (Total: 85,000 VH).

### 2D electrophoresis & mass spectrometry

SDS-PAGE followed IEF (per 2004 manual). Differentially abundant spots were excised and analyzed. Cytoplasmic proteins underwent LC-MS/MS (Institute of Microbiology). Sequences were aligned (KEGG/NCBI) with mouse, rat, zebrafish homologs to generate evolutionary trees.

### Interaction analysis

Gel-enzymolyzed pull-down samples (excluding MIC25 background) were compared to identify MIC25-interacting proteins via mass spectrometry.

### Yeast two-hybrid vector construction

MIC25’s ORF was cloned into BD-pGBKT7 (Clontech Gold Yeast Two-Hybrid System, PT3024−1). AP, CHCH3, and UBC ORFs (pull-down MS candidates) were inserted into AD-pGADT7. Primers are listed in Table 1.

### Yeast transformation & detection

Yeast competent cells (50 µL) were mixed with plasmid DNA (100 ng), carrier DNA (5 µL), and PEG/LiAc buffer (500 µL), incubated at 30°C (30 min), treated with DMSO (20 µL, 42°C, 15 min), centrifuged (13,000 rpm, 15 s), and resuspended in YPD medium (1 mL, 30°C, 1 h). Cells were plated on selective media, and colonies were PCR-verified. For β-gal assay, colonies were freeze-thawed (liquid nitrogen, 3–4×), immersed in β-buffer (Na2HPO4·7H2O 16.1 g/L, NaH2PO4·H2O 5.5 g/L, KCl 0.75 g/L, MgSO4·7H2O 0.246 g/L, pH 7.0), and incubated at 30°C (24–48 h).

### Luciferase Complementation (LCI)

ORFs were cloned into pcDNA3.1-nluc/cluc. Positive control: nluc-STG1A/cluc-RAR1 [25]. 293 cells were electroporated, cultured for 24 h, treated with D-luciferin (0.5 mM), and imaged via NightOwl (IB983) with 5s bright-field/5–10 min excitation.

### RNA extraction & RT

Muscle tissue (50 mg) was ground (liquid nitrogen), and RNA was extracted using RNeasy Micro Kit (Qiagen, 74106). Reverse transcription used SuperScript™ IV (Invitrogen, 18090010).

### RT-PCR & qPCR

RT-PCR used histone 3 as internal control (primers: Table 1), with products separated on 0.8% agarose gels. qPCR followed real-time PCR Master Mix (Toyobo, QPK-101) protocols on a CFX96 system (40 cycles).

### Nuclear/cytoplasmic protein extraction

Proteins were isolated using NE-PER™ Reagents (Thermo Scientific, 78333)

### Co-IP & western blot

Co-IP used Pierce™ Magnetic IP/Co-IP Kit (Thermo Scientific, 88828). Western blotting employed ECL (Thermo Scientific, 32209), with signals scanned conventionally.

### Ubiquitinated MIC25 electrophoresis

Ubiquitinated proteins were separated on 4–20% non-denaturing gels (Invitrogen) at 80V using Invitrogen buffers.

### Slow-twitch muscle force test

Isolated rodent slow-twitch muscles (tendons intact) were mounted on metal hooks (MDT, 820MO) in an oxygenated Organ Bath System. Microcurrent stimulation induced contractions; force was recorded at stable output (baseline-adjusted).

### Mitochondrial critical enzyme staining

Fresh mouse gastrocnemius muscles were sectioned and stained with MitoTracker™ Green FM (Invitrogen, M46750) diluted to 1 mM (Thermo Scientific, M7514), incubated (dark, 15 min), PBS-washed (×3), and stored at 4°C. Mitochondrial complexes I and V were stained using Cayman (700930) and Biotech (MBS9719098) kits, respectively. Reagents were diluted 1/10,000 in 1% BSA-PBS, washed post-staining, and stored at 4°C.

### Transwell chamber & ATP5MF detection

Cells (1 × 10⁵) were seeded into 15 cm Transwell grids with DMEM +10% FBS. After 24 h, KBM-2 medium was added below the grid. Post-4 days, elongated cells penetrating the mesh were fixed (4% PFA, 10 min), permeabilized (0.2% Tween-20, 2 min), and incubated with ATP5MF antibody (OriGene, AP20619PU-N; 1/5000 in PBST, 37°C, 2 h). Fluorescent secondary antibody (1/5000) was added (dark, 2 h), and bright-spot intensity was analyzed microscopically (10×).

### RNAi virus packaging & model

AAV9 (Addgene) vectors were inserted with MIC25/UBC3 ORFs via enzyme digestion/homologous recombination. Positive clones were sequenced and packaged (GenScript). Mice received caudal vein injections (1–5 × 10⁹ AAV9 particles in 10–50 µL) using a 100 µL microsyringe. RNAi was induced with tamoxifen 2 weeks post-injection.

### Caudal vein injection

Mice were restrained in a vented cylinder, tails pressed at a right-angle edge to expose veins. Veins were dilated (warm water/75% alcohol) before injecting AAV9 (muscle-specific) into dorsal/lateral caudal veins.

### MIC25 protein expression & purification

pET28a-MIC25-transformed BL21(DE3) cells were IPTG-induced, lysed, and purified via Ni-NTA (Invitrogen, K95001). Dialyzed protein was used for pull-down assays.

## Result

**Fig 1:**
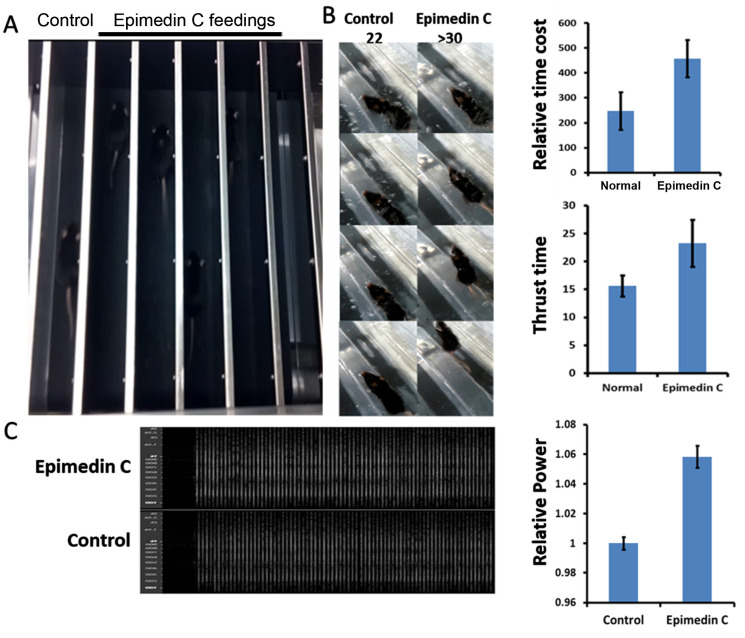
Endurance and explosive power test. A. Endurance test: Calculate running time under fixed speed conditions; The experimental group is mice fed Epimedin C, and the control group is mice fed with water solvent. B. Explosive force test. When mice swim normally at constant speed, sudden blistering on their tails causes convulsion. The picture shows statistics of the number of sprints after the convulsion. C. Comparison of relative power from isolated EDL muscle. The statistical graph shows the relative proportion of energy released after muscle contraction *P < 0.05, **P < 0.001.

Preliminary animal experiments with Epimedin C were administered by feeding, and administered to mouse models according to the dosage and method described in the Materials and Methods section. After a certain period of feeding and training, the mice’s exercise ability was measured using a treadmill measurement method. This was mainly to examine whether the muscles could continue to provide power and to observe the time period when fatigue occurs. As shown in Fig 1 A, the mouse treadmill experiment reflects that the average running time of mice fed Epimedin C is longer(>400min) than that of mice in the control group (200 ~ 300 min) without medication.

As shown in Fig 1B, in the mouse swimming experiment, at a constant speed, sudden bubbles are applied to the mouse tail from time to time. This is to make the mouse alert and accelerate swimming. What is tested is the mouse’s sprint power, and the number of times the mouse suddenly releases its sprint power. It was recorded that if the mouse did not jump significantly or swim faster under bubble hits, it was considered a negative result. The column shows the statistics of the mouse treadmill and swimming experiment. Compared to the nondrug group, the average running time of mice after Epimedin C feeding is about 2 times that of the drug-treated group, which is a difference but not significant.

The soleus muscle in the isolated muscle tissue is separated and the tendons on both sides are preserved. The tendons are hung in the probe in the test tank and stimulated with fixed-frequency electricity to collect kinetic energy data generated by muscle contraction. According to Fig 1C, the average kinetic energy output from the soleus muscle of mice treated with Epimedin C was greater than that of the control group of non-medicated mice (0.58%).

**Fig 2:**
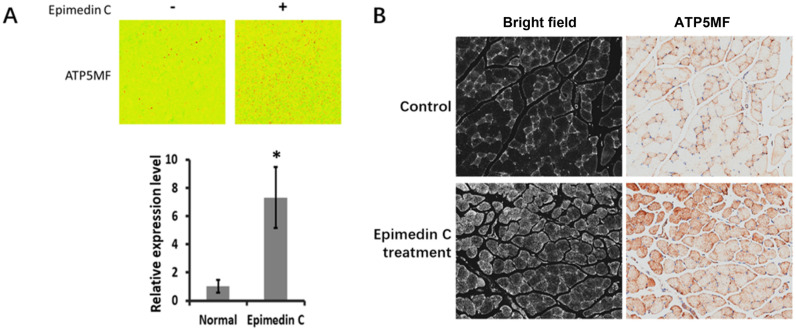
ATP related marker staining in SKM cells. A, ATP5MF were probed in isolated in rodent cells. *P < 0.05. B, SKM tissue staining by ATP5MF, bright field confirms the cells number as basic comparison group unit.

Examination in the subcellular microscopic field revealed differences in enzymes related to ATP synthesis. ATP5MF levels were higher in isolated muscles of mice fed Epimedin C compared with non-fed mice (Fig 2).

**Fig 3:**
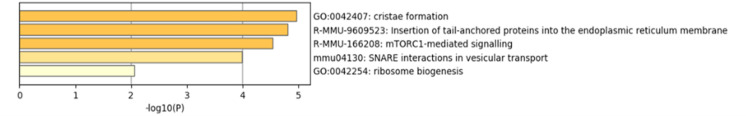
Results of proteome analysis. The cluster from proteome analysis shows differential proteins shown to be most relevant to energy metabolism.

After Epimedin C treatment, skeletal muscle tissue was isolated for mass spectrometry analysis. Cluster analysis of differential proteins showed that the mitochondrial ridge protein groups have obvious differences, indicating that the EC medication group has a significant abundance advantage. (Fig 3)

It is speculated that after Epimedin C medication, mice have significant changes in mitochondrial structure or function. Among many mitochondrial proteins, MIC25 has greater differences. Therefore, in the following experiments, MIC25 will be used as a priority candidate to analyze the relationship between Epimedin C and phenotype.

**Fig 4:**
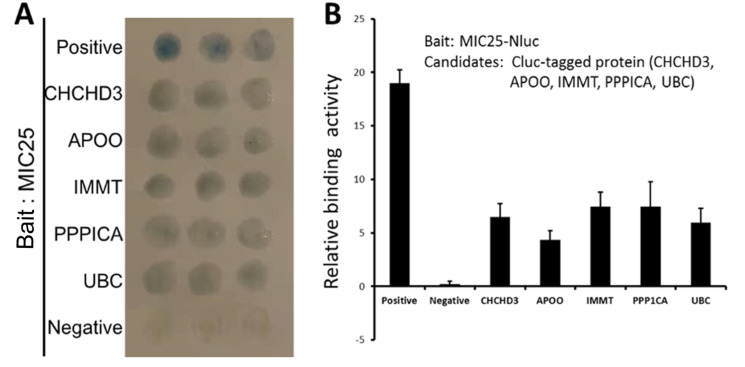
Yeast two-hybrid assay and luciferase complementation experiment. A, Yeast two-hybrid experiment, using MIC25 as bait protein mating test, the results after substrate color development; B, Insect luciferase complementation experiment, Nluc fusion-expressed MIC25 as bait, testing substrate fluorescence after protein interaction. Relative intensity and relative value *P < 0.05.

In subsequent experiments, this study used the MIC25 protein as bait and conducted yeast two-hybrid experiments to retrieve candidate proteins that may interact with it. Through yeast two-hybrid experiments, it was found that CHCHD3, APOO, IMMT, PPPICA, and a UBC family protein have significant binding effects (Fig 4).

**Fig 5:**
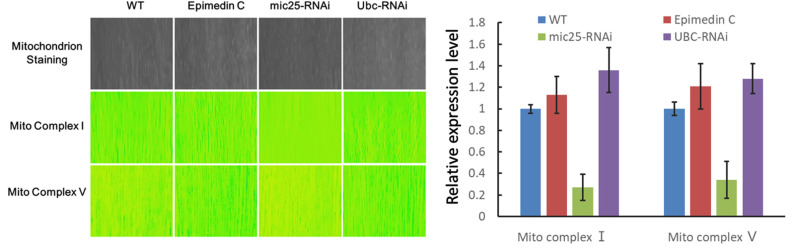
Staining of mitochondria and related enzymes in mouse skeletal muscle tissue. Staining results of mitochondria and related enzymes after Epimedin C and RNAi. Mitochondria staining is displayed in grayscale as the basic background and as an internal reference for fluorescence color.

In order to further verify the correlation between mitochondria and Epimedin C processing in the microscopic field, the energy-related components of mitochondria, Mito complex Ⅰ and Ⅴ, were stained on sections for observation. At the same time, to examine the impact of MIC25 and UBC on mitochondria, this study performed RNA silencing on the two genes respectively. As shown in Fig 5, after RNA silencing of MIC25, the Mito complex signal in muscle tissue got weakened, while the complex signal of UBC increased significantly after RNA silencing.

To further verify whether mitochondrial energy output is related to Epimedin C, this study used key enzymes related to ATP synthesis as targets for immunostaining and Western blot analysis.

**Fig 6:**
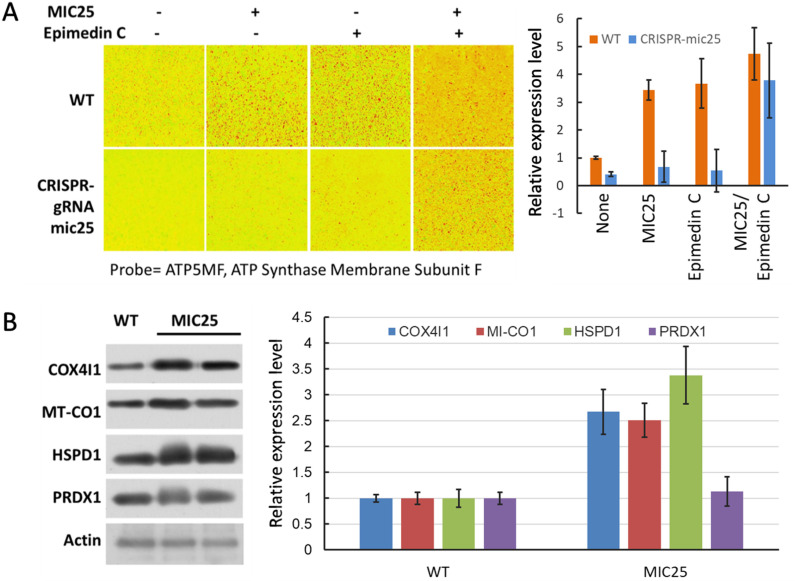
Relationship between energy related enzymes and MIC25 after Epimedin C treatment. A, Abundance of ATP5MF before and after treatment with MIC25 and Epimedin **C.** B, Relationship between energy-related enzyme content and MIC25 after Epimedin C treatment.

At the same time, exogenous MIC25 protein was used as an intervening factor in the immunostaining experiment. It was added to the system to observe the molecular phenotype. Fig 6 shows experimental results the application of exogenous MIC25 promoted the increase in ATP5MF enzyme content, and the application of Epimedin C also has a similar effect, while the simultaneous application of both has an additive effect. After MIC25 was silenced by RNAi, Epimedin C treatment did not increase ATP5F. Therefore, Epimedin C treatment may affect ATP5MF through MIC25. At the same time, COX4I1, MT-CO1, HSPD1, and PRDX1 were preliminarily tested in western blot experiments and found that MIC25 promotes the increase of these marker proteins.

**Fig 7:**
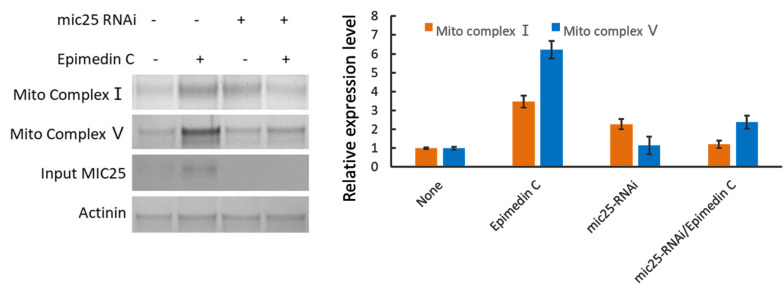
Epimedin C affects Mito Complex abundance through MIC25. MIC25 is used as an internal control in the system to show RNAi is effective.

After MIC25 was silenced by RNAi, Mito complexes Ⅰ and Ⅴ were significantly reduced, and Epimedin C treatment failed to rescue their abundance. It is speculated that Epimedin C affects Mito complex abundance through MIC25 (Fig 7).

**Fig 8:**
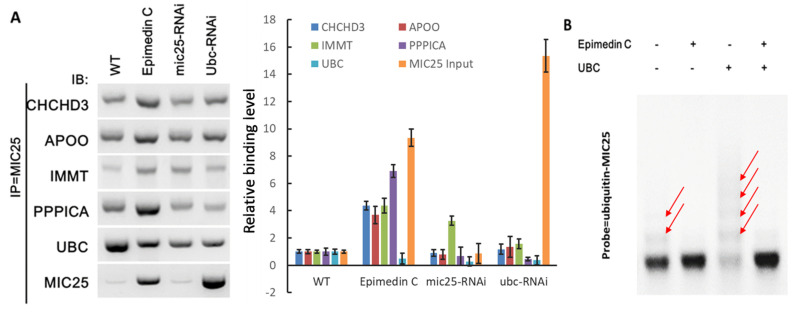
Effect of Epimedin C on MIC25 and its interacting proteins. A, CO-IP experiment to verify Epimedin C’s effect on MIC25 and its interacting proteins; B, the effect of Epimedin C on UBC ubiquitinated MIC25, ubiquitinated MIC25 is the target, and the top-down display after hybridization exhibits an increasing ladder-like signal.

In order to further verify the correlation between MIC25 interacting proteins and UBC, MIC25 was used as the bait protein in co-immunoprecipitation testing. The candidate proteins were all tested in WB experiments. Silencing MIC25 leads to a sharp reduction in candidate protein recruitment. The interaction signal is significantly weakened, as shown in Fig 8A. After the UBC protein was silenced by RNAi, MIC25 recruited more candidate proteins.

Since UBC protein adds ubiquitin to target proteins, this study further used a non-denaturing colloid as the electrophoresis matrix to separate ubiquitinated MIC25 and labeled it with a probe. Fig 8B shows exogenous UBC protein was added. It aggravated the ubiquitination of MIC25 protein (third lane in the Figure), showing an aggravated ladder-like signal. After Epimedin C treatment, this effect was effectively alleviated, and the ladder signal was significantly weakened. This result suggests that Epimedin C treatment may protect MIC25 from UBC-induced ubiquitination.

**Fig 9:**
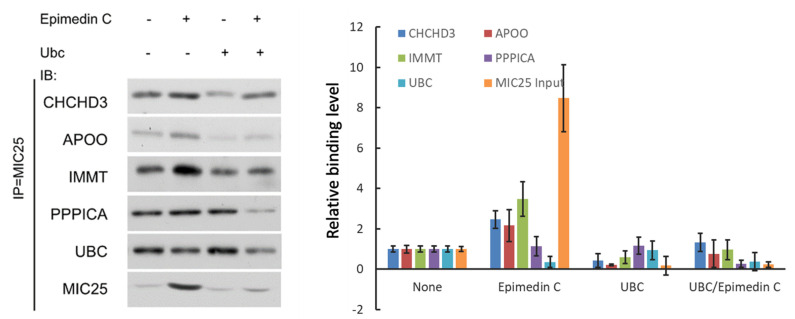
Changes in the interaction between MIC25 and its interacting respiratory chain proteins under treatment with Epimedin C and UBC. Antibody probes for MIC25 are used as a standard to confirm changes in MIC25 total amount.

At the same time, another set of co-immunoprecipitation experiments showed that Epimedin C treatment effectively alleviated the dissociation effect of UBC protein on MIC25 and other interacting proteins (Fig 9).

**Fig 10:**
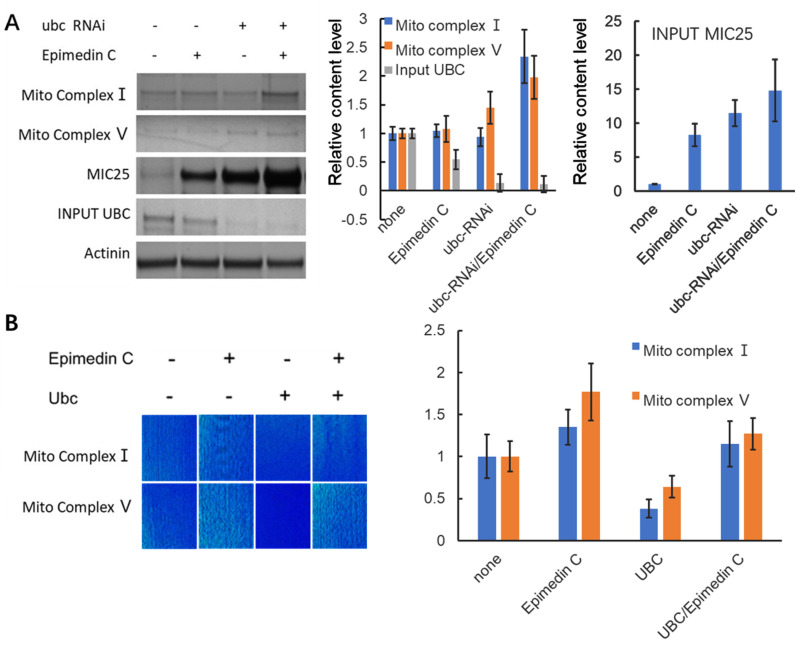
Epimedin C regulates the interaction of UBC and MIC25 to maintain the abundance of enzymes related to mitochondrial energy metabolism. A, Control group and RNAi treated with Epimedin C. Mito complex index under dual factors. The system internal parameter is input UBC to show RNAi effect. B, Mito complex index Mito Complex Ⅰ and Ⅴ in mice SKM, overexpressing UBC and control group treated with & without epimedin C.

In external models with UBC added, mitochondrial structure related markers Mito complex Ⅰ and Ⅴ were verified with the internal control of which Epimedin C were applied to in experiment. Fig 10 A shows Epimedin C antagonizes extra UBC effects and protects Mito complex Ⅰ and Ⅴ. The Mito complexes I and V in isolated muscles show almost the same effect (Fig 10 B).

**Fig 11:**
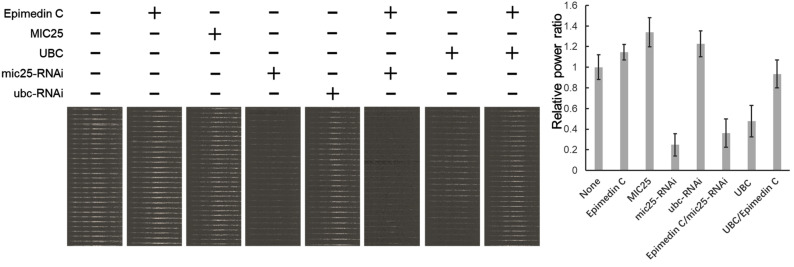
Power recorded from Isolated rodent EDL muscle under continuous microcurrent pulse stimulation. EDL muscle isolated from Epimedin C treated rodents whose MIC25 or UBC genes were silenced by RNAi. The groups with external MIC25 or UBC protein added ones were recorded in Organ Bath system (820MO) equipment.

This study investigates the role Epimedin C plays in the regulation of MIC25 and UBC. Isolated muscles were analyzed to measure force and power. The most direct evidence of Epimedin C’s ability to enhance athletic ability is shown in Fig 11. In the MIC25 silenced group, even adding additional Epimedin C failed to restore normal energy output in isolated muscles. This phenomenon shows that Epimedin C affects muscle energy output and requires MIC25 (Lane 6 in Fig 11). In the group that added UBC, it was found that Epimedin C could maintain almost normal energy output in isolated muscles (Lane 8 in Fig 11).

## Discussion

This study established cell and animal models to explore the effect of Epimedin C on mouse exercise ability and elucidate its mechanism.

To comprehensively evaluate the exercise-enhancing effects of Epimedin C, we conducted a multi-modal physiological assessment in rodents, integrating both *in vivo* behavioral tests and *ex vivo* biomechanical analyses. In Treadmill Exhaustion Test, A cohort of 24 C57BL/6 mice underwent graded treadmill running. Epimedin C-treated mice exhibited longer time-to-exhaustion compared to vehicle controls, demonstrating significant enhancement in sustained aerobic capacity. In Weight-Loaded Swimming Assay, Mice performed maximal-effort swimming with body weight. The Epimedin C group showed higher peak velocity during the initial burst phase, indicative of improved fast-twitch muscle energetics. Ex Vivo Contractile Energetics also conducted to mechanistically quantify energy output, we isolated slow-type soleus muscles (hindlimb deep compartment) and measured isometric force under microcurrent pulse stimulation (10 Hz, 500 ms trains). Epimedin C-treated muscles generated greater work output, confirming direct enhancement of neuromuscular efficiency. These macro-phenotypic improvements align with our molecular findings that Epimedin C preserves MIC25-dependent mitochondrial complex integrity, thereby optimizing ATP regeneration kinetics during both oxidative (treadmill) and glycolytic (swimming burst) energy demands.

Afterward, microscopic phenotyping assays examined key enzymes related to energy metabolism. This was to further illustrate the difference between energy differences and exercise phenotypes. In the chapter on mechanism research, we chose isolated muscle tissue as the material. We applied mass spectrometry to see which protein had a greater difference than the control group. Candidate proteins possess both Epimedin C-inducible properties and a direct correlation with phenotype. Later, interaction experiments and protein spectrum tests were conducted to determine that the candidate protein and its related proteins have a regulatory relationship. This relationship may be affected by Epimedin C, affecting the phenotype. Among the samples sent for mass spectrometry, there were 3 mice in each group. Follow-up experiments mainly used in vivo and in vitro cytology tests to verify that the interaction between MIC protein family members and UBC proteins changes with the participation of Epimedin C, thereby impacting the phenotype.

### 1. Epimedin C may enhance motor performance in mice by stabilizing the mitochondrial crest

In animal models, the exhaustion method was used at the drug treatment stage. Mice were forced to run with electric shock stimulation in the tail. The endurance of mice was assessed by recording exercise time under the condition of constant speed. At the same time, based on the uniform swimming pool experiment, the mice were frightened by the tail burst bubbles. The mice were forced to sprint, and the athletic explosive power of the mice was evaluated by recording the sprint speed [26]. In the treadmill experiment, mice in the Epimedin C treatment group performed more sustained exercise (>400min) than the control group (200 ~ 300 min). In the swimming pool experiment, mice treated with Epimedin C sprinted significantly more than those in the control group. That Epimedin C may improve exercise ability, which results in increased endurance and explosive power in mice.

### 2. Isolated muscle experiments confirm that Epimedin C contributes to the energy output of skeletal muscles

Electromyography is a direct method of assessing muscle power output [27].

To further determine the phenotype of skeletal muscle, electrical stimulation experiments on isolated muscles were designed. The long slow muscle of mouse legs was dissected as material. This material was mounted on the microprobe while providing nutrients and oxygen. The muscle contraction force transmitted by the probe was recorded by microcurrent. After this test, Epimedin C mice had an average energy output 5.8% higher than the control group. Because fast muscle energy output is more intense under micro-current stimulation, which is beyond the probe’s range, the waveform of isolated fast muscle was not recorded in this study. Based on this phenotype, our laboratory decided to discuss the functional impact and mechanism of Epimedin C on skeletal muscle as a topic.

### 3. Mass spectrometry screening confirms Epimedin C candidate targets

In order to elucidate the cause of this phenotype, fresh skeletal muscle samples were analyzed by mass spectrometry to obtain the most direct information. After mass spectrometry analysis, the mitochondrial protein MIC25 was significantly up-regulated after feeding. MIC25 was also up-regulated in human skeletal muscle cell culture, so it was speculated that this compound might be directly related to MIC25. Notes in the database showed that MIC25 was a member of the MICOS protein complex, belonging to the MIC protein family [28]. This is a group of proteins located in the mitochondrial crest. Previous studies have suggested that MICOS is a multi-subunit protein complex evolutionarily conserved in almost all species [29,30]. These proteins are mainly responsible for mitochondrial morphogenesis, maintain the normal development of mitochondrial crest, maintain the normal shape of mitochondrial crest, anchor respiratory chain related enzymes and mitochondrial outer membrane, and completely protect mitochondrial respiratory function within the structure [31]. Therefore, based on this information, we hypothesized that Epimedin C may improve mitochondrial function in the muscle model.

### 4. Epimedin C increases ATP5MF content through MIC25

When isolated cells were treated with Epimedin C, ATP5MF increased in the cells. In cells overexpressing MIC25, the enzyme also increased. The combined effect of the two showed an additive effect on ATP5MF. In cells with gRNA-mediated down-regulation of MIC25 expression, Epimedin C treatment failed to significantly up-regulate the enzyme, indicating that MIC25 plays a positive regulatory role in the up-regulation of ATP5MF caused by Epimedin C. It is upstream of ATP5MF and is affected by Epimedin C.

ATP5MF is a key enzyme related to ATP synthesis in mitochondria [32]. It is a crucial component of respiratory chain complex V, and its abundance is directly related to ATP production [33]. Based on the above-mentioned confirmation that Epimedin C can significantly enhance mice’ endurance and explosive power, it is speculated that ATP5MF under Epimedin C action is a key downstream node that ultimately affects energy output.

To quantitatively assess the metabolic consequences of UBC-MIC25 axis modulation, we measured intracellular ATP levels in gastrocnemius muscle homogenates. Strikingly, ATP content exhibited a strong positive correlation with MIC25 protein abundance, while showing an inverse relationship with UBC. Pharmacological intervention with Epimedin C not only elevated ATP levels in UBC-overexpressing cells but also abolished 89% of UBC-mediated ATP depletion (Supplement Fig 3), confirming its functional antagonism against UBC’s bioenergetic suppression.

This ATP restoration paralleled MIC25 stabilization and mitochondrial Complex I/V recovery suggesting a unified mechanism whereby Epimedin C preserves mitochondrial energy transduction through dual actions: Epimedin C inhibition of UBC-MIC25 binding and Downstream protection of MIC25-dependent complex assembly, thereby maintaining oxidative phosphorylation efficiency.

### 5. The candidate molecular interactions with MIC25 were found to respond to Epimedin C treatment

Epimedin C affects mitochondrial energy metabolism through MIC25 and UBC Database analysis shows that MIC25 is related to mitochondrial cristae structure, and the stability of mitochondrial cristae structure directly affects mitochondrial function and energy metabolism[34]. To prove that epimedin C can affect mitochondrial energy metabolism through MIC25 and UBC, we used isolated muscle tissue to stain key enzymes of energy metabolism in mitochondria and directly observed changes in energy metabolism histologically to verify the original hypothesis. This experiment still uses a mouse model. The RNAi viruses of MIC25 and UBC are packaged and injected into the body from the tail vein and suppressed. Living muscles are taken for mitochondrial staining as well as direct observation. Judging from the results, after epimedin C treatment, the mitochondrial staining signal was significantly higher than that of the control group. In contrast, the signal of MIC25-RNAi was significantly weakened, indicating that RNAi was effective, and UBC increased the mitochondrial signal after RNAi operation. This shows that epimedin C and MIC25 have positive effects on mitochondrial energy metabolism, while UBC has negative effects. In the same group of treatments, mitochondrial complex I and mitochondrial respiration-related complex V also showed similar trends. This experiment confirmed the effects of Epimedin C, MIC25 and UBC on mitochondrial number and energy metabolism from an intuitive tissue phenotype.

### 6. UBC binds to Mic25 and ubiquitinated later in skeletal muscle mitochondrial energy metabolism

The interaction between UBC and MIC25 was initially validated through co-immunoprecipitation (Co-IP) coupled with mass spectrometry (MS). To investigate ubiquitination dynamics, MIC25 protein was immunoprecipitated using a ubiquitin-specific antibody, revealing that epimedin C significantly attenuated MIC25 ubiquitination levels. Subsequent overexpression of exogenous UBC resulted in a marked increase in MIC25 ubiquitination, as evidenced by intensified ubiquitin-specific band shifts on Western blots (*e.g.*, higher molecular weight smears). This observation conclusively demonstrated that UBC directly mediates MIC25 ubiquitination.

To further delineate the role of epimedin C in this regulatory pathway, we examined its capacity to counteract UBC-driven ubiquitination. Strikingly, pretreatment with epimedin C effectively suppressed exogenous UBC-induced ubiquitination of MIC25. Collectively, these results establish that UBC serves as the primary ubiquitin ligase responsible for MIC25 ubiquitination, while epimedin C exerts its pharmacological effect by directly inhibiting UBC activity, thereby attenuating ubiquitination-dependent MIC25 degradation.

To investigate the regulatory effects of UBC and MIC25 abundance on mitochondrial complexes, we genetically or pharmacologically modulated UBC (overexpression) and MIC25 (knockdown) in skeletal muscle cells. Mitochondrial complexes were subsequently analyzed via immunoblotting using subunit-specific antibodies targeting Complex I (NDUFB8) and Complex V (ATP5A). Intriguingly, UBC upregulation combined with MIC25 downregulation synergistically reduced the protein levels of both Complex I and V. Notably, the suppression magnitude correlated with MIC25 depletion levels (*p* < 0.01), suggesting MIC25 is indispensable for maintaining mitochondrial complex integrity.

Co-immunoprecipitation (Co-IP) assays further revealed that Epimedin C treatment (10 μM, 24 h) disrupted the UBC-MIC25 interaction, as evidenced by diminished binding signals in Western blot analysis. Importantly, Epimedin C preserved MIC25 stability under UBC-overexpression conditions, thereby preventing downstream mitochondrial complex depletion.

These findings delineate a UBC-MIC25 regulatory axis governing mitochondrial bioenergetics: (1) UBC binds to and ubiquitinates MIC25, triggering its proteasomal degradation and subsequent collapse of energy-related enzyme complexes; (2) Epimedin C antagonizes this process by sterically blocking UBC-MIC25 binding, which stabilizes MIC25 and maintains mitochondrial complex homeostasis. This mechanism likely underlies Epimedin C’s capacity to enhance cellular ATP production in UBC-dysregulated models.

### 7. UBC and MIC25 are key molecules for Epimedin C to regulate mitochondrial enzyme activity and maintain mitochondrial structure

To validate the essential roles of UBC and MIC25 in Epimedin C-mediated preservation of mitochondrial enzyme activity and structural integrity, we employed siRNA-mediated knockdown of UBC and MIC25 in skeletal muscle cells and assessed mitochondrial structural markers and enzymatic activities. Notably, UBC knockdown induced a compensatory fold increase in MIC25 protein abundance, consistent with prior evidence that UBC directly promotes MIC25 ubiquitination and degradation. Concomitantly, mitochondrial Complex I and V protein levels increased respectively, confirming UBC’s negative regulatory role. Epimedin C treatment (20 μM, 48 h) synergistically enhanced this effect, further elevating Complex I/V abundance compared to UBC knockdown alone, suggesting additive stabilization of mitochondrial complexes.

To dissect MIC25’s indispensability, we performed MIC25 knockdown with/without Epimedin C co-treatment. MIC25 silencing reduced Complex I and V levels, confirming its critical role in maintaining mitochondrial complex homeostasis. Strikingly, Epimedin C failed to rescue Complex I/V depletion in MIC25-deficient cells, demonstrating absolute MIC25-dependency for its therapeutic efficacy.

These data establish a tripartite regulatory paradigm:

UBC functions as an E3 ubiquitin ligase targeting MIC25 for proteasomal degradation, thereby destabilizing mitochondrial complexes.

MIC25 serves as a non-redundant scaffold required for Complex I/V assembly and enzymatic competence.

Epimedin C exerts its mitochondrial-protective effect by antagonizing UBC-MIC25 interaction, thereby stabilizing MIC25 and preserving bioenergetic capacity

To directly assess the physiological impact of Epimedin C on energy metabolism, we performed *ex vivo* skeletal muscle contractility assays. These experiments provided critical functional validation of our molecular findings: Epimedin C treatment restored near-normal force output in UBC-overexpressing mice. This phenotypic rescue strongly supports our mechanistic model wherein Epimedin C preserves MIC25 stability by antagonizing UBC-mediated ubiquitination, as previously demonstrated through ubiquitin-specific Western blots. Consistent with this hypothesis, pharmacological protection of MIC25 correlated with preserved mitochondrial complex activities and ATP synthesis rates, suggesting its potential as a first-line intervention for UBC-related bioenergetic deficits.

**Fig 12:**
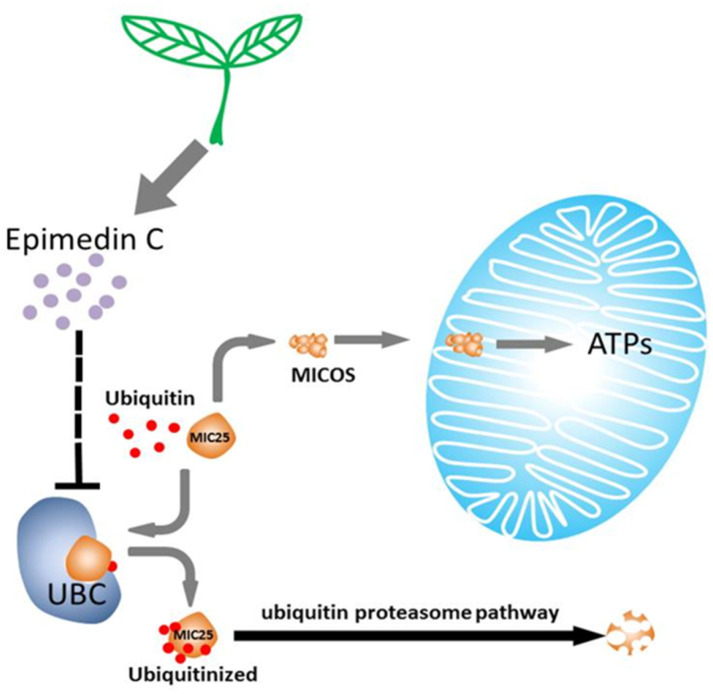
Epimedin C enhances the mitochondrial energy supply mechanism by regulating UBC and mic25 binding.

These functional gains extended to *in vivo* outcomes: Epimedin C-treated mice exhibited rescued exercise intolerance in rotarod tests. Collectively, our data establish Epimedin C a novel phytochemical agent, as both a molecular protector of MIC25 stability and a functional enhancer of striated muscle energetics through UBC-MIC25 axis modulation (Fig 12).

The correlation between Epimedin C and MIC25-UBC interaction is an encouraging start. Epimedin C interferes with the interaction between the two, suggesting that Epimedin C could be a new foreign aid intervention factor to treat abnormal symptoms related to MIC25-UBC interaction. A better application scenario may be the treatment of muscle fatigue. This is because abnormal mitochondrial energy supply is directly connected to muscle fatigue. The performance of the latter is similar to that of the mouse described in this article. Even markers related to muscle fatigue were not tested.

## Conclusion

Based on the above results, it was determined that MIC25 is the prerequisite for Epimedin C effect; UBC is the direct cause of MIC25 degradation, and UBC is the target affected by Epimedin C, which prevents UBC from ubiquitinating MIC25 and protects MIC25.

This study discovered that Epimedin C inhibits the interaction of UBC and MIC25, protecting the latter from degradation, thereby maintaining the stability of the MICOS complex, ensuring the integrity of mitochondrial functional units, and providing material support for muscle cells to provide sufficient ATP, making the muscle organizations exhibit better athletic ability. This article may have reference significance for future muscle fatigue treatment.

### Limitation

It could be argued that MIC25 may not be the only molecular modulated by Epimedin C, considering the limitations of MS assay and protein interaction library. Other molecular components of SKM may not be excluded in future laboratory works. Another limitation of this article is that the method in the study was not sufficient to explore the direct downstream molecules of Epimedin C and the MIC25/UBC relationship. The effect of this flavonoid on MIC25 and UBC is a clue, and more upstream molecules have yet to be discovered.

## Supporting information

Supplementary Fig 1 Epimedin C feeding concentration and serum testosterone concentration. (TIF)

Supplementary Fig 2 A, Mic25 transcription levels after Epimedin C treatment. B, Mic25 evolution tree analysis in different animal model. (TIF)

Supplementary Fig 3 ATP assays. (TIF)

## References

[1] Benaroya H. Mitochondria and MICOS – function and modeling. Reviews in the Neurosciences. 2024;35: 503–31. doi: 10.1515/revneuro-2024-0004 38369708

[2] Casanova A, Wevers A, Navarro-Ledesma S, Pruimboom L. Mitochondria: It is all about energy. Front Physiol. 2023;14:1114231. doi: 10.3389/fphys.2023.1114231 37179826 PMC10167337

[3] Baker N, Patel J, Khacho M. Linking mitochondrial dynamics, cristae remodeling and supercomplex formation: How mitochondrial structure can regulate bioenergetics. Mitochondrion. 2019;49:259–68. doi: 10.1016/j.mito.2019.06.003 31207408

[4] Caron C, Bertolin G. Cristae shaping and dynamics in mitochondrial function. J Cell Sci. 2024;137(1):jcs260986. doi: 10.1242/jcs.260986 38197774

[5] Di Marco G, Gherardi G, De Mario A, Piazza I, Baraldo M, Mattarei A, et al. The mitochondrial ATP-dependent potassium channel (mitoKATP) controls skeletal muscle structure and function. Cell Death Dis. 2024;15(1):58. doi: 10.1038/s41419-024-06426-x 38233399 PMC10794173

[6] Wculek SK, Heras-Murillo I, Mastrangelo A, Mañanes D, Galán M, Miguel V, et al. Oxidative phosphorylation selectively orchestrates tissue macrophage homeostasis. Immunity. 2023;56(3):516–530.e9. doi: 10.1016/j.immuni.2023.01.011 36738738

[7] Kao Y, Chou C-H, Huang L-C, Tsai C-K. Momordicine I suppresses glioma growth by promoting apoptosis and impairing mitochondrial oxidative phosphorylation. EXCLI J. 2023;22:482–98. doi: 10.17179/excli2023-6129 37534227 PMC10391611

[8] Ul Fatima N, Ananthanarayanan V. Mitochondrial movers and shapers: Recent insights into regulators of fission, fusion and transport. Curr Opin Cell Biol. 2023;80:102150. doi: 10.1016/j.ceb.2022.102150 36580830

[9] von der Malsburg A, Sapp GM, Zuccaro KE, von Appen A, Moss FR 3rd, Kalia R, et al. Structural mechanism of mitochondrial membrane remodelling by human OPA1. Nature. 2023;620(7976):1101–8. doi: 10.1038/s41586-023-06441-6 37612504 PMC10875962

[10] Hong J, Guan X, Chen Y, Tan X, Zhang S, Feng G. Mitochondrial Membrane Potential Independent Near-Infrared Mitochondrial Viscosity Probes for Real-Time Tracking Mitophagy. Anal Chem. 2023;95(13):5687–94. doi: 10.1021/acs.analchem.2c05568 36940187

[11] Mühleip A, Flygaard RK, Baradaran R, Haapanen O, Gruhl T, Tobiasson V, et al. Structural basis of mitochondrial membrane bending by the I-II-III2-IV2 supercomplex. Nature. 2023;615(7954):934–8. doi: 10.1038/s41586-023-05817-y 36949187 PMC10060162

[12] Ding C, Wu Z, Huang L, Wang Y, Xue J, Chen S, et al. Mitofilin and CHCHD6 physically interact with Sam50 to sustain cristae structure. Sci Rep. 2015;5:16064. doi: 10.1038/srep16064 26530328 PMC4632003

[13] Huang C, Deng K, Wu M. Mitochondrial cristae in health and disease. Int J Biol Macromol. 2023;235:123755. doi: 10.1016/j.ijbiomac.2023.123755 36812974

[14] Stephan T, Brüser C, Deckers M, Steyer AM, Balzarotti F, Barbot M, et al. MICOS assembly controls mitochondrial inner membrane remodeling and crista junction redistribution to mediate cristae formation. EMBO J. 2020;39(14):e104105. doi: 10.15252/embj.2019104105 32567732 PMC7361284

[15] Kozjak-Pavlovic V. The MICOS complex of human mitochondria. Cell Tissue Res. 2017;367(1):83–93. doi: 10.1007/s00441-016-2433-7 27245231

[16] Li P, Zhang L, Guo Z, Kang Q, Chen C, Liu X, et al. Epimedium koreanum Nakai-Induced Liver Injury-A Mechanistic Study Using Untargeted Metabolomics. Front Pharmacol. 2022;13:934057. doi: 10.3389/fphar.2022.934057 35910368 PMC9326364

[17] Liu Y, Zhang H, Liu Y, Chen Y. Active constituents and mechanism of Epimedii folium against liver cancer: a review. Chin J Exp Tradit Med Formulae. :217–25.

[18] Liu T-Z, Chen C-Y, Yiin S-J, Chen C-H, Cheng J-T, Shih M-K, et al. Molecular mechanism of cell cycle blockage of hepatoma SK-Hep-1 cells by Epimedin C through suppression of mitogen-activated protein kinase activation and increased expression of CDK inhibitors p21(Cip1) and p27(Kip1). Food Chem Toxicol. 2006;44(2):227–35. doi: 10.1016/j.fct.2005.07.003 16112786

[19] Huang M, Yu L, Wang Y, Yang C. Epimedin C protects dexamethasone-induced osteoblasts through NRF1/RhoA pathway. Aging. 2023;15(6):2033–45. doi: 10.18632/aging.204588 36920182 PMC10085613

[20] Gang R, Nagarajan SM, Anandhan P. RETRACTED ARTICLE: Mechanism of the effect of traditional Chinese medicine fumigation on blood lactic acid in exercise body. J Ambient Intell Human Comput. 2020;12(3):3295–301. doi: 10.1007/s12652-020-02356-6

[21] Kumari S, Singh M, , Jain S, Verma N, Malik S, et al. A review on therapeutic mechanism of medicinal plants against osteoporosis: effects of phytoconstituents. Mol Biol Rep. 2023;50(11):9453–68. doi: 10.1007/s11033-023-08751-4 37676432

[22] Lin Y-A, Li Y-R, Chang Y-C, Hsu M-C, Chen S-T. Activation of IGF-1 pathway and suppression of atrophy related genes are involved in Epimedium extract (icariin) promoted C2C12 myotube hypertrophy. Sci Rep. 2021;11(1):10790. doi: 10.1038/s41598-021-89039-0 34031457 PMC8144409

[23] Tafuri A, Sebben M, Shakir A, Pirozzi M, Processali T, Rizzetto R, et al. Endogenous testosterone mirrors prostate cancer aggressiveness: correlation between basal testosterone serum levels and prostate cancer European Urology Association clinical risk classes in a large cohort of Caucasian patients. Int Urol Nephrol. 2020;52(7):1261–9. doi: 10.1007/s11255-020-02398-x 32016908

[24] Unerwood W, Raymond A. AVMA Guidelines for the Euthanasia of Animals: 2020 Edition.

[25] Chen H, Zou Y, Shang Y, Lin H, Wang Y, Cai R, et al. Firefly luciferase complementation imaging assay for protein-protein interactions in plants. Plant Physiol. 2008;146(2):368–76. doi: 10.1104/pp.107.111740 18065554 PMC2245818

[26] Islam H, Edgett BA, Bonafiglia JT, Shulman T, Ma A, Quadrilatero J, et al. Repeatability of exercise-induced changes in mRNA expression and technical considerations for qPCR analysis in human skeletal muscle. Exp Physiol. 2019;104(3):407–20. doi: 10.1113/EP087401 30657617

[27] Rampichini S, Vieira TM, Castiglioni P, Merati G. Complexity Analysis of Surface Electromyography for Assessing the Myoelectric Manifestation of Muscle Fatigue: A Review. Entropy (Basel). 2020;22(5):529. doi: 10.3390/e22050529 33286301 PMC7517022

[28] Vue Z, Garza-Lopez E, Neikirk K, Katti P, Vang L, Beasley H, et al. 3D reconstruction of murine mitochondria reveals changes in structure during aging linked to the MICOS complex. Aging Cell. 2023;22(12):e14009. doi: 10.1111/acel.14009 37960952 PMC10726809

[29] Busch JD, Fielden LF, Pfanner N, Wiedemann N. Mitochondrial protein transport: Versatility of translocases and mechanisms. Mol Cell. 2023;83(6):890–910. doi: 10.1016/j.molcel.2023.02.020 36931257

[30] Khosravi S, Harner ME. The MICOS complex, a structural element of mitochondria with versatile functions. Biol Chem. 2020;401(6–7):765–78. doi: 10.1515/hsz-2020-0103 32229686

[31] Maruyama T, Hama Y, Noda NN. Mechanisms of mitochondrial reorganization. J Biochem. 2024;175(2):167–78. doi: 10.1093/jb/mvad098 38016932

[32] Sadeesh EM, Lahamge MS, Malik A, Ampadi AN. Differential Expression of Nuclear-Encoded Mitochondrial Protein Genes of ATP Synthase Across Different Tissues of Female Buffalo. Mol Biotechnol. 2025;67(2):705–22. doi: 10.1007/s12033-024-01085-x 38305843

[33] Vercellino I, Sazanov LA. The assembly, regulation and function of the mitochondrial respiratory chain. Nat Rev Mol Cell Biol. 2022;23(2):141–61. doi: 10.1038/s41580-021-00415-0 34621061

[34] Rampelt H, Wollweber F, Licheva M, de Boer R, Perschil I, Steidle L, et al. Dual role of Mic10 in mitochondrial cristae organization and ATP synthase-linked metabolic adaptation and respiratory growth. Cell Rep. 2022;38(4):110290. doi: 10.1016/j.celrep.2021.110290 35081352 PMC8810396

