# Supplementary figures and images for: Epimedin C enhances mitochondrial energy supply by regulating the interaction between MIC25 and UBC in rodent model
Source: PLoS One. 2025 May 28;20(5):e0325031. doi: 10.1371/journal.pone.0325031 (PMC12119004; doi:10.1371/journal.pone.0325031)

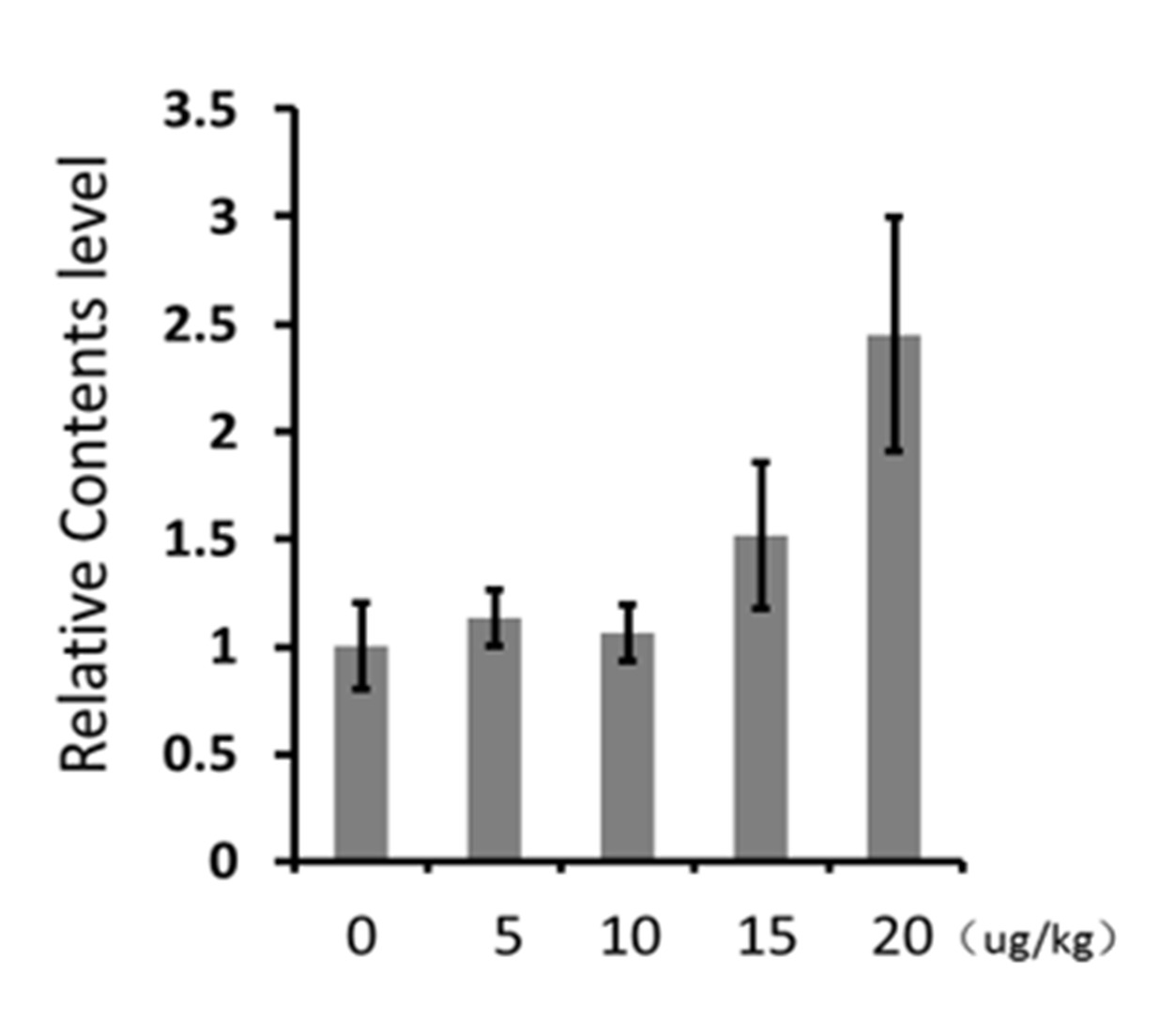

Supplement: Supplementary Fig 1 — (TIF) [file pone.0325031.s001.tif]

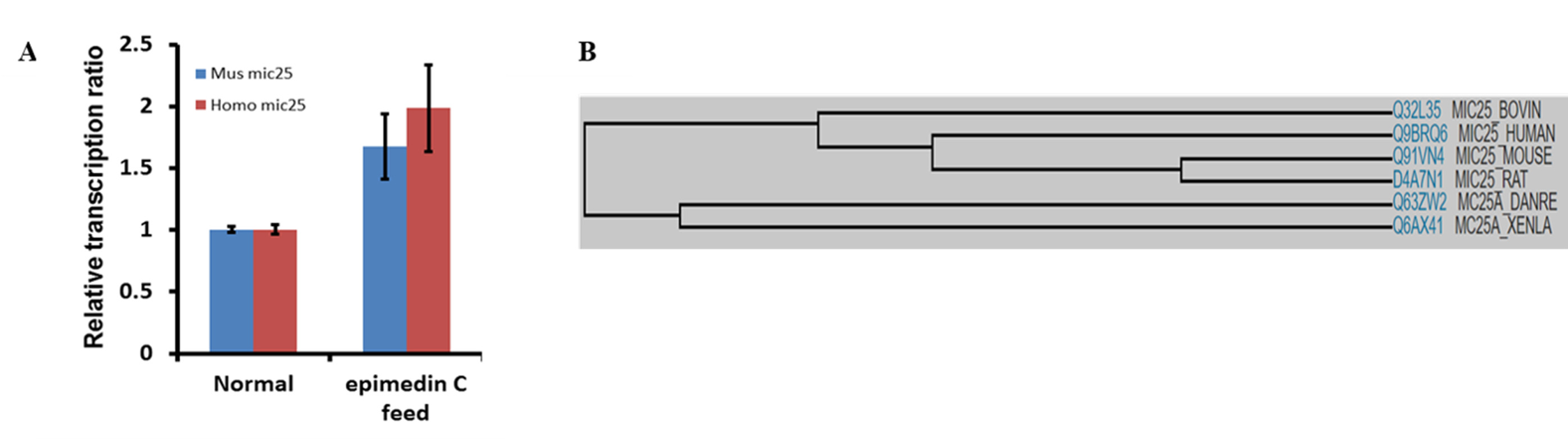

Supplement: Supplementary Fig 2 — (TIF) [file pone.0325031.s002.tif]

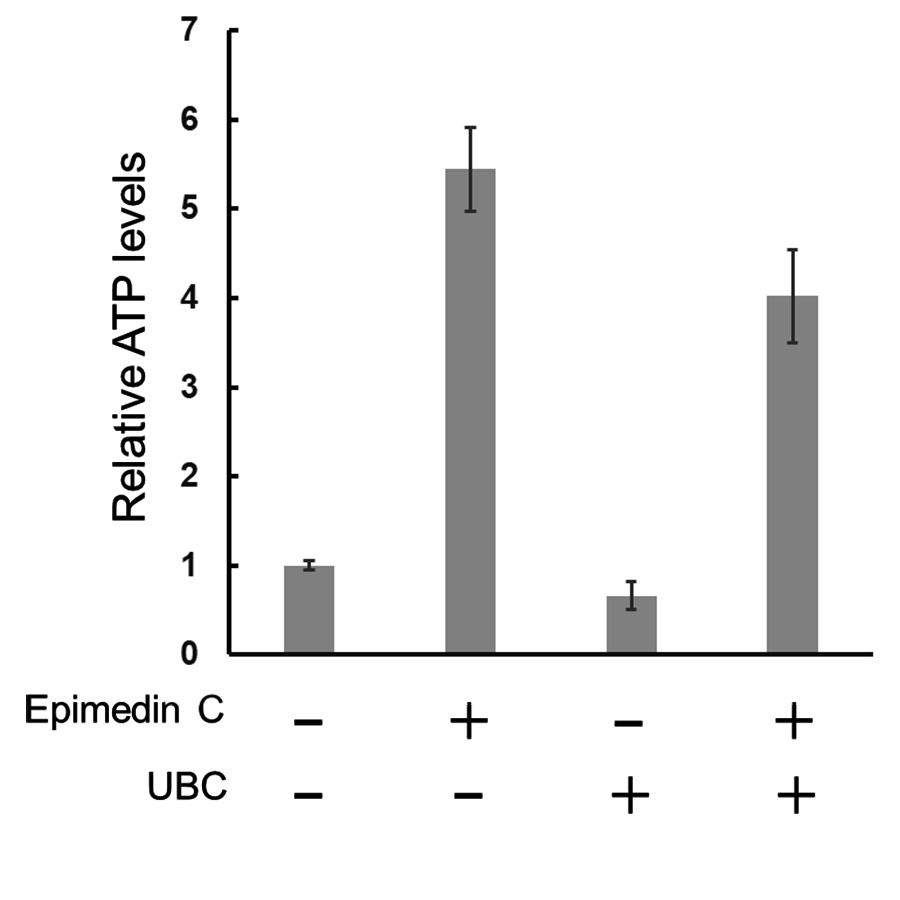

Supplement: Supplementary Fig 3 — (TIF) [file pone.0325031.s003.tif]
